# Dietary Nitrate Supplementation Enhances Performance and Speeds Muscle Deoxyhaemoglobin Kinetics during an End-Sprint after Prolonged Moderate-Intensity Exercise

**DOI:** 10.3390/antiox12010025

**Published:** 2022-12-23

**Authors:** Samantha N. Rowland, Mariasole Da Boit, Rachel Tan, George P. Robinson, Emma O’Donnell, Lewis J. James, Stephen J. Bailey

**Affiliations:** 1 School of Sport, Exercise and Health Sciences, Loughborough University, Loughborough LE11 3TU, UK; 2 Health and Life Sciences, School of Allied Health Sciences, De Montfort University, Leicester LE1 9BH, UK; 3 Department of Sports Medicine, Pepperdine University, Malibu, CA 90263, USA

**Keywords:** nitric oxide, beetroot powder, exercise performance, near-infrared spectroscopy

## Abstract

Short-term dietary nitrate (NO_3_^−^) supplementation has the potential to enhance performance during submaximal endurance, and short-duration, maximal-intensity exercise. However, it has yet to be determined whether NO_3_^−^ supplementation before and during submaximal endurance exercise can improve performance during a short-duration, maximal-intensity end-sprint. In a randomised, double-blind, crossover study, 9 recreationally active men ingested NO_3_^−^-rich (BR: 8 mmol NO_3_^−^/day) and NO_3_^−^-depleted (PL: 0.75 mmol NO_3_^−^/day) beetroot powder for 7 days. On day 7, participants completed 2 h of moderate-intensity cycling, which immediately transitioned into a 60 s maximal-intensity end-sprint, with supplements ingested 2 h before and 1 h into the moderate-intensity exercise bout. Plasma [NO_3_^−^] and [NO_2_^−^] were higher in BR compared to PL pre- and post-exercise (*p* < 0.05). Post-exercise plasma [NO_3_^−^] was higher than pre-exercise (562 ± 89 µM vs. 300 ± 73 µM; *p* < 0.05) and plasma [NO_2_^−^] was not significantly different pre- (280 ± 58 nM) and post-exercise (228 ± 63 nM) in the BR condition (*p* > 0.05). Mean power output during the final 30 s of the end-sprint was greater after BR (390 ± 38 W) compared to PL (365 ± 41 W; *p* < 0.05). There were no differences between BR and PL in any muscle oxygenation variables during moderate-intensity cycling (*p* > 0.05), but muscle [deoxyhaemoglobin] kinetics was faster during the end-sprint in BR (6.5 ± 1.4 s) compared to PL (7.3 ± 1.4 s; *p* < 0.05). These findings suggest that NO_3_^−^ supplementation has the potential to improve end-sprint performance in endurance events when ingested prior to and during exercise.

## 1. Introduction

Dietary supplementation with inorganic nitrate (NO_3_^−^) has emerged as a strategy to bolster performance in a variety of exercise settings in recreationally active individuals [1]. The ergogenic effects of NO_3_^−^ ingestion have been attributed to the stepwise reduction of NO_3_^−^ to nitrite (NO_2_^−^) and, subsequently, NO_2_^−^ to nitric oxide (NO) [2]. It is now recognised that NO_3_^−^ is absorbed in the upper gastrointestinal tract after ingestion with ~25% of circulating NO_3_^−^ actively taken up by the salivary glands and concentrated in saliva [3,4]. Anaerobic bacteria in the oral cavity then reduce salivary NO_3_^−^ to NO_2_^−^ [5]. Once swallowed, a portion of this NO_2_^−^ is further reduced to NO and other reactive nitrogen intermediates (RNIs), such as S-nitrosothiols (RSNO), in the stomach, with the remainder entering the systemic circulation, increasing plasma [NO_2_^−^], as well as [RSNO] [6,7]. More recent evidence also suggests skeletal muscle [NO_3_^−^] and [NO_2_^−^] can be increased following dietary NO_3_^−^ supplementation and that skeletal muscle is an important NO_3_^−^ storage organ [2,8,9]. Subsequently, circulating systemic and local muscle [NO_3_^−^] and [NO_2_^−^] can be used as substrates for O_2_ independent NO synthesis [2,8], with the potential to modulate an array of physiological processes [2,10,11].

Initial studies reported improved performance during continuous, submaximal endurance exercise after dietary NO_3_^−^ ingestion [1,12,13], with NO_3_^−^ supplementation now appearing to confer greater ergogenic potential for shorter duration higher intensity (<15 min), compared to longer duration lower intensity (>1000 s), endurance exercise [1]. Consistent with this observation, an initial study reported that dietary NO_3_^−^ supplementation did not improve 50 mile cycling time trial (TT) performance (>120 min completion time); however, a negative correlation was observed between the increase in plasma [NO_2_^−^] and the lowering of 50 mile TT completion time after NO_3_^−^ ingestion [14]. Accordingly, these data suggest greater potential for an ergogenic effect during longer duration endurance exercise in individuals presenting with higher plasma [NO_2_^−^] after NO_3_^−^ ingestion. More recently, Tan et al. [15] reported that NO_3_^−^ ingestion prior to and after 1 h of a 2 h moderate-intensity cycling task prevented the decline in plasma [NO_2_^−^], and blunted the progressive increases in the oxygen cost of exercise and muscle glycogen depletion, compared with placebo ingestion during exercise and when NO_3_^−^ was only consumed pre-exercise. However, and despite better maintenance of plasma [NO_2_^−^] and improved metabolic efficiency after NO_3_^−^ supplementation prior to and during exercise, performance in a subsequent 100 kJ cycling TT was not improved [15]. A necessary limitation of this study [15] was an interruption between the end of the moderate-intensity exercise bout and transition into the TT performance test to obtain muscle biopsy samples to assess metabolic responses. Such an interruption does not typically reflect real life competitive scenarios and may have permitted partial recovery of perturbations to muscle phosphocreatine (PCr), inorganic phosphate (Pi) and adenosine diphosphate (ADP), which are known to recover exponentially following exercise cessation [16]. Since NO_3_^−^ supplementation has been reported to attenuate perturbation to these intramuscular phosphorous substrates and metabolites concomitant with enhanced exercise tolerance [12], this may account for the lack of an ergogenic effect in this previous study by Tan et al. [15]. It is also possible that, since the increase in muscle [NO_3_^−^] after NO_3_^−^ supplementation declines during exercise [9] and increases rapidly (within 30 min) following acute NO_3_^−^ ingestion [17], an in-exercise NO_3_^−^ top-up bolus can better maintain the local increases in muscle [NO_3_^−^] and [NO_2_^−^] achieved after pre-exercise NO_3_^−^ ingestion. Therefore, further research is required to evaluate the ergogenic efficacy of an in-exercise NO_3_^−^ top-up dose for improving prolonged endurance performance.

Analysis of pacing strategies indicates that adopting a relatively even pacing strategy for the majority of the race followed by an end-of-race sprint (end-sprint) or ‘kick’ is commonly applied in competitive endurance events [18]. Although NO_3_^−^ supplementation has been reported to improve muscle contractile function in a non-fatigued state [19,20,21], there is evidence to suggest that NO_3_^−^ supplementation may be more likely to improve neuromuscular function after completing fatigue-inducing exercise [22]. Similarly, while NO_3_^−^ supplementation has been reported to improve time to peak power output, peak power output and/or mean power output during maximal sprints initiated from an unfatigued state in some studies [23,24,25,26], it has yet to be determined whether these findings are reproducible when the sprint is commenced after a prolonged period of moderate-intensity endurance exercise.

As prolonged, submaximal exercise unfolds, there is a progressive recruitment of fast-twitch (type II) muscle fibres to maintain the required force production, as some of the initially recruited slow-twitch (type I) muscle fibres become substrate depleted and fatigued [27]. It is also well documented that the proportional recruitment of type II muscle fibres is positively associated with exercise intensity [28,29,30]. Accordingly, greater type II muscle fibre recruitment would be expected during the latter stages of an endurance event, particularly when terminated with an end-sprint. It is established that PO_2_ and pH are lowered to a greater extent in contracting type II relative to type I muscle [31,32], and that hypoxia and acidosis facilitate both NO_2_^−^ reduction to NO [33,34] and NO_3_^−^ reduction to NO_2_^−^ in skeletal muscle [35]. As such, the physiological and metabolic conditions evoked by a prolonged period of moderate-intensity endurance exercise terminated with a maximal end-sprint has the potential to elicit an ergogenic effect after NO_3_^−^ supplementation, but this postulate has not been experimentally tested. Moreover, it has previously been reported that muscle [deoxyhaemoglobin + deoxymyoglobin] ([HHb]) kinetics was faster, suggestive of enhanced muscle fractional O_2_ extraction during high-intensity exercise initiated after 4 min of moderate-intensity cycling exercise, concomitant with improved time to exhaustion [36]. However, it has yet to be determined whether [HHb] kinetics is altered during an end-sprint completed after prolonged moderate-intensity cycling exercise.

The purpose of the present study was to investigate whether ingestion of NO_3_^−^-rich beetroot powder before and during 2 h of moderate-intensity cycling influenced plasma [NO_3_^−^] and [NO_2_^−^], and end-sprint performance compared to a placebo condition. In addition, near-infrared spectroscopy (NIRS) was used to assess local muscle (de)oxygenation variables to provide physiological insight into any performance enhancement conferred by NO_3_^−^ supplementation. It was hypothesized that NO_3_^−^ supplementation before and during 2 h moderate-intensity exercise would: (1) further increase plasma [NO_3_^−^] and preserve elevated [NO_2_^−^]; (2) speed [HHb] kinetics; and (3) improve end-sprint performance.

## 2. Materials and Methods

### 2.1. Participant Characteristics

Nine recreationally active male participants (mean ± SD: age: 21 ± 1 years, stature: 1.81 ± 0.06 m, body mass: 77.4 ± 12.5 kg, $\dot{V}$O_2peak_: 49.0 ± 5.1 mL·kg^−1^·min^−1^) completed the study protocol. None of the participants were tobacco smokers. All experimental procedures were approved by Loughborough University Research Ethics Approvals Human Participants Sub Committee. Participants were fully informed of the risks and discomforts associated with all trials before providing written, informed consent. Participants were instructed to arrive at each laboratory testing session in a rested and hydrated state, having avoided strenuous exercise in the 24 h prior to each visit. Each participant was given a list of NO_3_^−^-rich foods to avoid consuming 24 h before testing sessions and asked to avoid caffeine and alcohol ingestion 12 h and 24 h before each trial, respectively. Participants were instructed to record their diet 24 h prior to their first experimental test and were asked to replicate this prior to each subsequent test. For the duration of the study, participants were asked to abstain from using antibacterial mouthwash since it blunts NO_3_^−^ reduction to NO_2_^−^ in the oral cavity [37].

### 2.2. Experimental Design

Participants reported to the laboratory on four occasions. During the first visit, participants completed a ramp incremental cycling test for determination of gas exchange threshold (GET), peak aerobic power (PAP) and peak oxygen uptake ($\dot{V}$O_2peak_). During the second visit, participants performed a 60 s all-out sprint on the cycle ergometer and were familiarised with the moderate-intensity exercise work rate. Thereafter, participants were assigned to receive two separate 7 day supplementation periods with a beetroot powder (TruBeet, Bio-gen Extracts, Bangalore, India) that was either NO_3_^−^-rich (BR) or NO_3_^−^-depleted (PL) as part of a randomised, cross-over experimental design. During the two main experimental testing sessions, which were completed on day 7 of the supplementation periods, participants completed 2 h of moderate-intensity cycling exercise which immediately transitioned into a 60 s end-sprint performance test. Muscle oxygenation variables were assessed, heart rate (HR) and ratings of perceived exertion (RPE) were determined, and venous blood samples were taken. All exercise testing was performed at the same time of day (±2 h) for each participant.

### 2.3. Incremental Test

During the first visit, participants completed a ramp incremental test on an electronically braked cycle ergometer (Lode Excalibur Sport, Groningen, The Netherlands). Initially, participants completed 4 min of baseline cycling at 20 W, after which the work rate increased linearly by 30 W/min until task failure. Task failure was recorded once the pedal rate fell ≥10 rpm below the participant’s self-selected cadence (70–90 rpm) for 5 s. The saddle and handlebar height and configuration were recorded and reproduced in subsequent tests. Breath-by-breath pulmonary gas exchange data were collected continuously (Vyntus CPX metabolic cart, Vyaire Medical, Chicago IL, USA) and averaged over consecutive 10 s periods. Participants wore a face mask and breathed through a low dead space, low resistance, digital volume transducer assembly. The inspired and expired gas volume and gas concentration signals were continuously sampled via a capillary line connected to the mouthpiece. The gas analyser was calibrated prior to testing with gases of known concentration. The turbine volume transducer was calibrated automatically and manually using a 3 L syringe (Hans Rudolph, Kansas City, MO, USA). $\dot{V}$O_2peak_ was taken as the highest 30 s mean value attained prior to volitional exhaustion. GET was determined from a cluster of measurements including (1) the first disproportionate increase in CO_2_ production ($\dot{V}$CO_2_) from visual inspection of individual plots of $\dot{V}$CO_2_ vs. $\dot{V}$O_2_, (2) an increase in minute ventilation ($\dot{V}$_E_)/$\dot{V}$O_2_ with no increase in $\dot{V}$_E_/$\dot{V}$CO_2_, and (3) an increase in end-tidal O_2_ tension with no fall in end-tidal CO_2_ tension. Work rates that would elicit 90% of GET (moderate-intensity exercise) and 50%Δ (GET plus 50% of the difference between power output at GET and PAP) were subsequently calculated, with two-thirds of the ramp rate deducted from the work rate at GET and PAP to account for the $\dot{V}$O_2_ mean response time.

### 2.4. Familiarisation Test

Participants completed a 60 s all-out sprint against a fixed and constant resistance. The resistance on the pedals was set using the cadence-dependent linear mode on the cycle ergometer so that participants attained a power output calculated to be 50%Δ upon attaining their self-selected cadence from the initial incremental test (linear factor = power output/preferred cadence^2^). Participants were provided with a 5 s countdown prior to the sprint and were instructed to attain peak power as quickly as possible and to continue exercising maximally for the duration of the effort. Participants were also familiarised with the moderate-intensity exercise work rate. This involved 2 min cycling at 25 W followed by a step increase in work rate equivalent to 90% GET for 10 min.

### 2.5. Supplementation Procedure

After the initial ramp test and familiarisation visits, participants were assigned to receive supplementation with BR containing 6% NO_3_^−^ (8 mmol NO_3_^−^) or PL containing 0.56% NO_3_^−^ (0.75 mmol NO_3_^−^) in a randomised, counter-balanced order. The PL powder was prepared by manipulating the processing control parameters (extraction time and temperature) during extract preparation to ensure that NO_3_^−^ was not being completely extracted and was degraded/removed during the process. Specifically, raw beetroot was extracted with water for 1 h at >90 °C with the residue obtained from the first extraction processed to obtain the low NO_3_^−^ grade material. The NO_3_^−^ content of BR and PL were determined by treating the samples with a salicylic acid-sulphuric acid mixture, and subsequently 2 N sodium hydroxide, before measuring absorbance using a UV-Vis spectrophotometer (UV-1800, Shimadzu, Japan) with values compared to potassium NO_3_^−^ standards. On days 1–6 of the supplementation periods, participants consumed 8.4 g of TruBeet powder mixed with ≥250 mL of water. On day 7 of supplementation, when the participants underwent the experimental testing procedures, participants were instructed to ingest the TruBeet powder 2 h before reporting to the laboratory to coincide with peak plasma [NO_2_^−^] [38]. In addition, 1 h into the exercise trials, participants were also given a top-up 8.4 g dose of TruBeet powder (PL or BR) in 250 mL water. Supplementation periods were separated by a minimum of 10 days washout and were administered double-blind.

### 2.6. Experimental Tests

After arrival at the laboratory and attachment of the NIRS device, participants rested in a supine position for 10 min before a venous blood sample was collected. Participants then commenced the exercise protocol. This consisted of 4 min baseline cycling at 25 W followed by 2 h cycling at 90% GET. HR and RPE were recorded every 10 min during the exercise test. The exercise bouts then immediately transitioned into a 60 s all-out sprint. Following completion of the exercise, participants rested supine for 10 min before a venous blood sample was taken.

### 2.7. Measurements

#### 2.7.1. Sprint Performance

Power output was recorded continuously at 5 Hz using the Lode Excalibur Sport software and data were subsequently exported, reduced to second-by-second values and analysed to derive time to peak power output, peak power output and mean power outputs between 0–60 s, 0–30 s and 30–60 s.

#### 2.7.2. Muscle Oxygenation

Changes in the oxygenation status of the *m. vastus lateralis* of the right leg during the protocol were continuously assessed using NIRS (PortaMon, Artinis Medical Systems, Einsteinweg, The Netherlands). Initially, the area surrounding the muscle belly was shaved and cleaned. The NIRS probe was affixed over the midway point between the superior border of the iliac crest and fibular head using kinesiology tape. Elastic strapping was then used to secure the device in place and minimise the possibility of extraneous light influencing the signal. The exact location of the device was recorded to enable precise reproduction of the placement in subsequent tests. The NIRS device emitted near-infrared light from 3 diodes at 760 and 850 nm, at distances of 30, 35 and 40 mm from the receiver. Data were sampled at 10 Hz and recorded with Oxysoft software (Artinins, Netherlands). During offline analysis, data were exported and averaged to 1 s intervals for later analysis. Relative changes in tissue saturation index (TSI) and concentrations of oxyhaemoglobin + oxymyoglobin (O_2_Hb), deoxyhaemoglobin + deoxymyoglobin (HHb) and total haemoglobin + myoglobin were determined.

To provide information on muscle deoxygenation kinetics, the [HHb] response to exercise was fitted with a monoexponetial model. Specifically, the [HHb] kinetics during the end-sprint bouts were determined by fitting a monoexponential model from the first data point 1 SD above the baseline mean through to the point at which the response departed from a monoexponentially, as determined from the residual plot and described in the following equation:

HHb (t) = HHbbaseline + A (1 − e ^− (t−TD/τ)^)

where HHb (t) represents the relative HHb at a given time t; HHbbaseline represents the mean HHb in the baseline period; A, TD, and τ represent the amplitude, time delay, and time constant, respectively, describing the increase in HHb above baseline. The [HHb] TD and τ values were summed to provide information on the overall [HHb] response dynamics. It has been reported that NIRS kinetics is a reliable method to assess muscle oxidative capacity following moderate-intensity exercise [39]. The [HHb] signal was used to provide an estimate of fractional O_2_ extraction in the area under interrogation. TSI, calculated as:

TSI (%) = [O_2_Hb]/([O_2_Hb] + [HHb]) × 100

provides a percentage oxygenation in the area under interrogation.

#### 2.7.3. Ratings of Perceived Exertion and Heart Rate

Both RPE, using the 6–20 scale, and HR (Polar monitor, Kempele, Finland) were recorded every 10 min during the exercise tests.

#### 2.7.4. Plasma [NO_3_^−^] and [NO_2_^−^]

Venous blood samples were drawn from an antecubital vein into 6 mL lithium-heparin tubes (Sarstedt, Leicester, UK) at baseline and post exercise. Samples were centrifuged at 3500× *g* for 10 min at 4 °C, within 3 min of collection. Plasma was subsequently extracted and immediately frozen at −80 °C for later analysis of [NO_3_^−^] and [NO_2_^−^].

### 2.8. Data Analysis Procedures

#### [NO_3_^−^] and [NO_3_^−^] Determination

All glassware, utensils and surfaces were rinsed with deionised water to remove residual NO prior to [NO_3_^−^] and [NO_2_^−^] analysis. Plasma samples were deproteinised using zinc sulfate (ZnSO_4_)/sodium hydroxide (NaOH) precipitation prior to [NO_3_^−^] determination. Firstly, 250 μL of 0.36 M NaOH was added to 50 μL of sample followed by 5 min incubation at room temperature. Subsequently, samples were treated with 150 μL of aqueous ZnSO_4_ (10% *w*/*v*) and vortexed for 30 s before undergoing an additional 10 min incubation period at room temperature. Samples were then centrifuged at 17,000× *g* for 8 min and the supernatant was removed for subsequent analysis. The [NO_3_^−^] of the deproteinised plasma sample was determined by its reduction to NO in the presence of 0.8% (*w*/*v*) vanadium chloride in 1 M hydrochloric acid within an air-tight purging vessel. The spectral emission of electronically excited nitrogen dioxide, derived from the reaction of NO with ozone, was detected by a thermoelectrically cooled, red-sensitive photomultiplier tube house in a Sievers gas-phase chemiluminescence nitric oxide analyser (Sievers NOA 280i, Analytix Ltd., Durham, UK). The [NO_3_^−^] was determined by plotting signal (mV) against a calibration plot of sodium NO_3_^−^ standards. Plasma samples were deproteinised using ice-cold ethanol precipitation prior to [NO_2_^−^] determination. Specifically, 500 μL of sample was treated with 1000 μL ice-cold ethanol followed by 30 min incubation on ice. Samples were then centrifuged at 17,000× *g* for 8 min and the supernatant was removed for subsequent analysis. The [NO_2_^−^] of deproteinised plasma was determined by its reduction to NO in the presence of glacial acetic acid and aqueous sodium iodide (4% *w*/*v*) and calibrated using sodium NO_2_^−^ standards.

### 2.9. Statistical Analysis

Two way (time × supplement) repeated-measures ANOVAs were used to assess changes in RPE, HR, and plasma [NO_3_^−^] and [NO_2_^−^]. Significant effects were followed up with post hoc paired *t*-tests with Holm-Bonferroni correction. Paired *t*-tests were used to assess changes in sprint performance and NIRS variables during the end-sprint. All data are presented as mean ± SD unless otherwise stated. Effect size (ES) for the ANOVAs were calculated using partial eta squared with ES for paired *t*-tests calculated as Cohen’s dz (*t*/√n). Statistical analysis was performed using IBM SPSS Statistics version 27. Statistical significance was accepted at *p* < 0.05.

## 3. Results

The PL and BR supplements in this study were well tolerated with no adverse side effects reported. Participants were unable to distinguish between the PL and BR supplements.

### 3.1. Plasma [NO_3_^−^] and [NO_2_^−^]

There were main effects for supplement (ES = 0.97) and time (ES = 0.98), and a supplement × time interaction effect (ES = 0.96) for plasma [NO_3_^−^] (all *p* < 0.001). Plasma [NO_3_^−^] was higher pre- (ES = 3.23) and post-exercise (ES = 5.91) in BR compared to PL (all *p* < 0.001, Table 1). There was no difference between plasma [NO_3_^−^] pre-to-post exercise in the PL condition (*p* > 0.05, ES = 1.08), but post-exercise plasma [NO_3_^−^] was 87% higher than pre-exercise plasma [NO_3_^−^] in the BR condition after receiving the top-up BR dose (*p* < 0.001, ES = 5.46). There were main effects for supplement (*p* < 0.001, ES = 0.86) and time (*p* < 0.05, ES = 0.47), but no supplement × time interaction effect for plasma [NO_2_^−^] (*p* > 0.05, ES = 0.15). Plasma [NO_2_^−^] was higher pre- (ES = 3.04) and post-exercise (ES = 0.993) in BR compared to PL but was not different between time points within the BR (ES = 0.63) and PL (ES = 0.22) conditions (all *p* < 0.05, Table 1).

### 3.2. Sprint Performance

**Figure 1:**
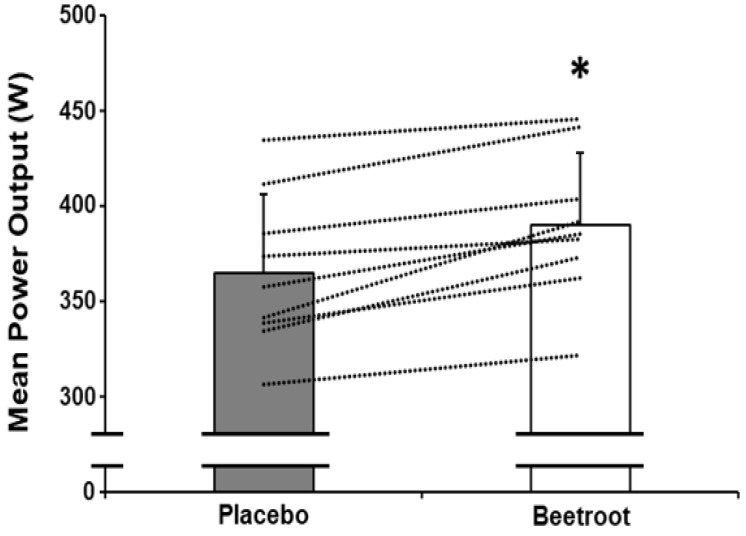
Mean power output between 30–60 s of a maximal 60 s end-sprint following 2 h moderate-intensity exercise after ingestion of nitrate-depleted beetroot (placebo, grey bar) and nitrate-rich beetroot (beetroot, open bar) powder. Dashed lines represent individual responses. Data presented as mean ± SD. * indicates significantly higher than placebo (*p* < 0.001).

**Figure 2:**
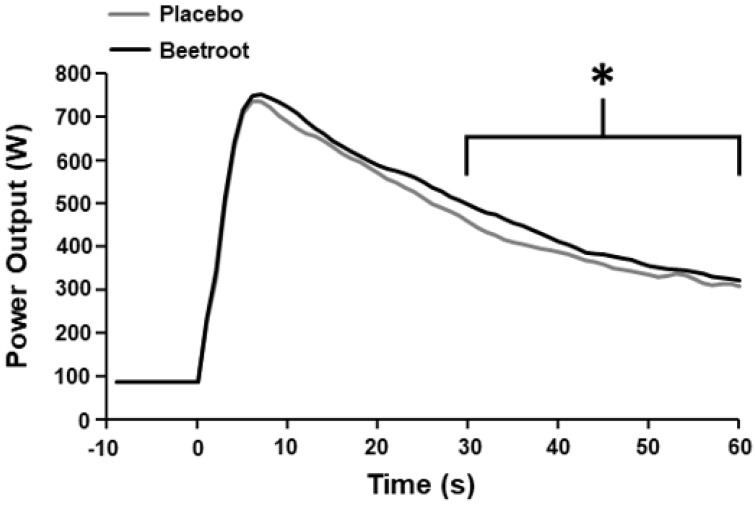
Mean power output during the 60 s end-sprint following 2 h moderate-intensity exercise after ingestion of nitrate-depleted beetroot (placebo) and nitrate-rich beetroot (beetroot) powder. * Higher than placebo (*p* < 0.001). Error bars are omitted for clarity.

Time to peak power output (*p* = 0.49, ES = 0.24) and peak power output (*p* = 0.61, ES = 0.18) did not differ between PL and BR (Table 2). Mean power output between 0–60 s was not significantly different between PL and BR (*p* = 0.058, ES = 0.74); Table 2). However, mean power output was greater in the BR condition between 30–60 s (*p* < 0.01, ES = 1.82; Figure 1 and Figure 2).

### 3.3. Muscle Oxygenation

**Figure 3:**
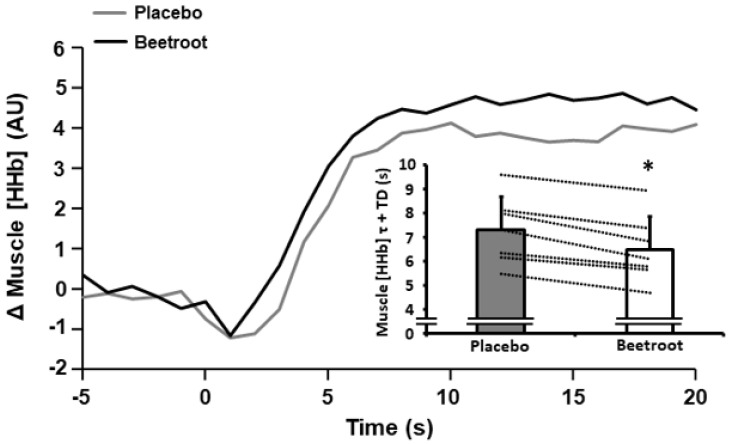
Muscle deoxyhaemoglobin + deoxymyooglobin concentration ([HHb]) response profiles during the initial stages of a 60 s end-sprint following 2 h moderate-intensity exercise after ingestion of nitrate-depleted beetroot (placebo) and nitrate-rich beetroot (beetroot) powder in a representative individual. Data are expressed as the change (Δ) from baseline. Inset illustrates muscle [HHb] time constant (τ) + time delay (TD) after ingestion of placebo (grey bar) and beetroot (open bar) powder. Dashed lines represent individual responses. Data presented as mean ± SD. * indicates significantly lower than placebo (*p* < 0.001).

After initial inspection, NIRS data from 7 of 9 participants was of sufficient quality for analysis and was used in the current paper. There were no between-condition differences in TSI during 2 h moderate-intensity cycling exercise or during the end-sprint (*p* > 0.05; Table 3). However, during the end-sprint muscle [HHb] τ + TD was lower in the BR compared to the PL condition (6.5 ± 1.4 s vs. 7.3 ± 1.4 s; *p* < 0.001, ES = 2.50; Figure 3).

### 3.4. Ratings of Perceived Exertion and Heart Rate

There was a main effect of time (*p* < 0.001), but no effect of supplement (*p* > 0.05), or time × supplement interaction (*p* > 0.05) for RPE and HR (data not reported).

## 4. Discussion

Ingestion of NO_3_^−^ prior to and 1 h into 2 h of moderate-intensity cycling culminating in a 60 s end-sprint, increased post-exercise compared to pre-exercise plasma [NO_3_^−^] and blunted the decline in plasma [NO_2_^−^] that typically occurs when NO_3_^−^ is only consumed before exercise [15,40]. In addition, NO_3_^−^ consumption before and during exercise speeded muscle [HHb] kinetics over the initial stages, and improved mean power output over the latter stages, of the 60 s end-sprint. These observations are consistent with our experimental hypotheses and suggest that short-term NO_3_^−^ supplementation for 6 days, coupled with acute NO_3_^−^ ingestion 2 h prior to and 1 h into a 2 h moderate-intensity exercise bout on day 7, has the potential to improve end-sprint performance. These findings may have implications for recreationally active participants aiming to enhance prolonged endurance exercise performance via dietary NO_3_^−^ supplementation.

### 4.1. Effect of BR Supplementation on Plasma [NO_3_^−^] and [NO_2_^−^]

Plasma [NO_3_^−^] and [NO_2_^−^] were elevated pre-exercise following 7 days of NO_3_^−^ supplementation with the final 8 mmol NO_3_^−^ consumed 2 h prior to exercise, compared to PL. These findings are in line with previous research which has consistently reported elevations in both plasma [NO_3_^−^] and [NO_2_^−^] following similar dietary NO_3_^−^ supplementation protocols [12,13,15]. The pharmacokinetic responses to acute dietary NO_3_^−^ ingestion are well described, with peak plasma [NO_3_^−^] and [NO_2_^−^] expected to be, respectively obtained ~1 h and 2–3 h post ingestion of 8 mmol NO_3_^−^ [38]. Plasma [NO_3_^−^] is relatively stable during exercise, at least up to 1 h, after NO_3_^−^ ingestion [15]. In the present study, participants consumed an additional 8 mmol NO_3_^−^ top-up bolus 1 h into a 2 h moderate-intensity cycle bout which was terminated with a 60 s maximal sprint with venous blood sampled 10 min thereafter. Compared to pre-exercise values after NO_3_^−^ supplementation, the ingestion of an 8 mmol NO_3_^−^ top-up bolus during exercise elicited a further increase in plasma [NO_3_^−^], with no changes between the pre-and post-exercise values after PL supplementation, consistent with the previous observations by Tan et al. [15]. In contrast, ingestion of an 8 mmol NO_3_^−^ top-up bolus did not increase plasma [NO_2_^−^] post-exercise compared to pre-exercise values. Tan et al. [15] also observed no increase in post-exercise compared to pre-exercise plasma [NO_2_^−^] after ingestion of a NO_3_^−^ top-up bolus 1 h into 2 h of moderate-intensity exercise followed by a 100 kJ cycling TT; conversely, these authors observed lower post-exercise compared to pre-exercise plasma [NO_2_^−^] in this condition as well as NO_3_^−^ supplementation without the additional top-up bolus. While plasma [NO_2_^−^] is relatively stable during moderate-intensity exercise after NO_3_^−^ ingestion [15,40], plasma [NO_2_^−^] has been reported to decline during high-intensity exercise in numerous studies [15,40,41,42]. The differences in the plasma [NO_2_^−^] dynamics between the present study and the previous study by Tan et al. [15] may be attributed to inter-study differences in the supplementation procedures and exercise testing protocol. Specifically, an 8 mmol NO_3_^−^ top-up bolus was administered in the current study with 6 mmol administered in the study of Tan et al. [15], and the 2 h moderate-intensity cycle stage was followed by a 60 s maximal sprint in the current study compared to a longer 100 kJ cycling TT (completion time of ~400 s) in the study of Tan et al. [15]. Therefore, an important original contribution of the present study is that the ingestion of an 8 mmol NO_3_^−^ top-up bolus can attenuate the decline in plasma [NO_2_^−^] that occurs during high-intensity exercise after only ingesting NO_3_^−^ pre-exercise.

### 4.2. Effect of BR Supplementation on Maximal End-Sprint Performance

Compared to PL, 7 days of 8 mmol·day^−1^ NO_3_^−^ supplementation with an 8 mmol top-up NO_3_^−^ bolus consumed 1 h into a 2 h moderate-intensity cycle bout that was terminated with a 60 s maximal sprint, improved mean power output over the final 30 s of the sprint. To our knowledge, these are the first data to support enhanced performance after short-term NO_3_^−^ supplementation with the consumption of an in-exercise NO_3_^−^ top-up bolus. In a previous study by Tan et al. [15], 3 days of 12.4 mmol·day^−1^ NO_3_^−^ supplementation with a 6.2 mmol top-up NO_3_^−^ bolus consumed 1 h into a 2 h moderate-intensity cycle bout terminated with a 100 kJ cycling TT, did not enhance performance in the TT. These disparities may be linked to differences in supplementation procedures, performance test, and the interlude between the completion of the 2 h moderate-intensity exercise and commencement of the TT in the study of Tan et al. [15] to enable muscle biopsy sampling. Indeed, dietary NO_3_^−^ intake was increased for 7 days versus 3 days, and the in-exercise NO_3_^−^ top-up dose was 8 mmol versus 6 mmol in the present study versus the study by Tan et al. [15], respectively. Therefore, the greater total duration and dose of NO_3_^−^ administered in the days preceding the performance test, and the greater in-exercise NO_3_^−^ top-up dose may have contributed to the ergogenic effects reported in the current study and not in the study by Tan et al. [15]. It is also possible that the greater degree of skeletal muscle hypoxia and acidosis [12,40,43], and greater recruitment of type II muscle fibres [28,29,30], that would be expected during a maximal-intensity 60 s sprint versus a lower intensity longer duration TT test may be more conducive for NO_2_^−^ reduction to NO [33,34], which may account for the greater ergogenic effect of NO_3_^−^ supplementation in the present study compared to the previous study by Tan et al. [15]. Furthermore, the experimental protocol used by Tan et al. [15] incorporated a rest period between the moderate-intensity exercise bout and TT for muscle biopsy sampling, which may have permitted partial PCr, ADP and Pi recovery. Since NO_3_^−^ supplementation may be ergogenic, at least in part through attenuating perturbations to muscle [PCr], [ADP] and [Pi], such effects may have been lost prior to commencing the TT in the study of Tan et al. [15] given the exponential recovery of these molecules [16], thereby limiting the ergogenic potential of NO_3_^−^ supplementation in that study.

Although performance was improved over the final 30 s of the sprint in the present study, time to peak power output, peak power output and mean power output over the entire 60 s sprint did not differ between PL and BR. These results conflict with some, but not all, studies reporting enhanced sprint performance after NO_3_^−^ supplementation when the sprint is completed in the absence of a 2 h moderate-intensity exercise pre-load [23,24,25,26]. While neuromuscular fatigue was not determined after 2 h moderate-intensity cycling exercise in the current study, neuromuscular fatigue development has been observed following extended moderate-intensity exercise [44]. Therefore, our observations are consistent with previous findings indicating that NO_3_^−^ supplementation may be particularly effective at enhancing muscle function after exercise-induced neuromuscular fatigue [22]. The finding of enhanced performance over the second half, but not the first half, of the 60 s end-sprint is also consistent with this interpretation.

### 4.3. Effect of BR Supplementation on Muscle Oxygenation Variables

There was no effect of NO_3_^−^ supplementation on NIRS-derived TSI during the moderate-intensity exercise bout in the current study. This observation conflicts with some, but not all, previous reports of improved lower limb muscle oxygenation during moderate-intensity exercise following dietary NO_3_^−^ supplementation [13,40,41]. While muscle TSI was not altered during the 60 s end-sprint, HHb τ + TD was lower (faster) after NO_3_^−^ supplementation. Faster muscle HHb kinetics in the transition from moderate-intensity to higher intensity, constant work rate, submaximal exercise after NO_3_^−^ supplementation has previously been reported by Breese et al. [36]; however, our observations extent these previous findings to suggest that such effects can also occur after prolonged moderate-intensity exercise that transitions into a maximal-intensity end-sprint. Muscle [HHb] kinetics has been suggested to offer a non-invasive proxy for fractional muscle O_2_ extraction during exercise [45]. In the previous study by Breese et al. [36], faster muscle [HHb] kinetics (determined via frequency-domain NIRS) after NO_3_^−^ supplementation was accompanied by faster pulmonary $\dot{V}$O_2_ kinetics, a non-invasive surrogate for muscle $\dot{V}$O_2_ kinetics [46]. While it is recognised that a limitation of the current study is the lack of assessment of pulmonary gas exchange variables, it is possible that faster muscle [HHb] kinetics after NO_3_^−^ supplementation facilitated faster $\dot{V}$O_2_ kinetics. Since time to peak power output, peak power output and mean power over the first 30 s of the 60 s sprint did not differ between PL and BR, faster [HHb] kinetics were not a function of greater power output or cadence over the initial stages of the sprint [47]. Expedited muscle [HHb] kinetics may, therefore, have increased the proportional oxidative energy turnover, with a corresponding reduction in anaerobic energy turnover, to facilitate enhanced performance over the latter stages of the 60 s end-sprint. Indeed, such metabolic changes would be expected to lower the decline in muscle PCr utilisation and ADP and Pi accumulation, which contribute to neuromuscular fatigue development [48] and have been previously reported after NO_3_^−^ supplementation [12].

### 4.4. Experimental Considerations and Implications

Improved performance during a 60 s end-sprint following 2 h moderate-intensity exercise after NO_3_^−^ supplementation in the current study may have implications for improving race performance during longer duration endurance events. Indeed, it has been reported that a common pacing strategy during longer duration endurance events is a relatively even pace for the majority of a race with increased speed over the latter stages of the event. Specifically, over 10,000 m to half-marathon running events, most studies indicate that the optimal pacing strategy is a relatively even-pace with the race terminating in an all-out end-sprint [49]. Such pacing strategies are also employed to great effect by “sprinters” to win some Grand Tour cycling stages. As such, through improving end-sprint performance, NO_3_^−^ supplementation has potential to improve performance outcomes in settings where a prolonged bout of relatively evenly paced endurance exercise is terminated with an end-sprint. However, it is acknowledged that alternative pacing strategies can be employed to deliver successful race outcomes, such as adopting a relatively constant, but higher mean race pace, or completing stochastic periods of increased mean race pace before returning to a relatively even race pace. Further research is required to assess the ergogenic potential of NO_3_^−^ supplementation in such settings. Moreover, since better trained endurance athletes seem less receptive to an ergogenic effect of acute and short-term NO_3_^−^ supplementation on endurance performance [50,51], further research is required to address whether NO_3_^−^ supplementation prior to and during longer duration endurance exercise can elicit an ergogenic effect in this population.

Whilst the data presented in the current study suggest that NO_3_^−^ supplementation prior to and during 2 h moderate-intensity exercise can improve end-sprint performance concomitant with faster muscle [HHb] kinetics, better maintenance of plasma [NO_2_^−^] and a further increase in plasma [NO_3_^−^], there are some limitations with the current study that are recognised. Firstly, the small sample size is acknowledged as a limitation of the current study. This may have precluded the detection of improved mean power over the whole 60 s end-sprint (*p* = 0.058). In addition, venous blood was only sampled pre- and post-exercise, and as such, it is unclear whether plasma [NO_2_^−^] was increase after 2 h moderate exercise following consumption of the NO_3_^−^ top-up at 1 h of the moderate exercise protocol compared to pre-exercise values. It is also recognised that, since the current study did not evaluate the independent effects of short-term NO_3_^−^ supplementation with the last dose ingested 2 h prior to exercise, or acute NO_3_^−^ supplementation prior to and during exercise, it is unclear whether these NO_3_^−^ supplementation strategies may have been comparatively ergogenic to the one adopted in the current study. However, the findings of the present study, combined with the previous study by Tan et al. [15] which did isolate the effect of consuming an in-exercise NO_3_^−^ top-up, collectively support a NO_3_^−^ top-up supplementation strategy to improve physiological and performance responses during longer duration endurance exercise.

Faster muscle [HHb] kinetics at the start of the end-sprint after NO_3_^−^ supplementation in the current study should be interpreted with some caution as data were collected using a continuous-wave NIRS system. However, our findings are consistent with previous observations of faster muscle [HHb] kinetics in the transition from moderate-intensity to higher-intensity exercise after NO_3_^−^ supplementation when using a superior, frequency-domain NIRS system [36]. In addition, while pre-exercise plasma [NO_3_^−^] was increased after NO_3_^−^ supplementation, an in-exercise NO_3_^−^ top-up after 1 h moderate-intensity exercise elicited a further increase in plasma [NO_3_^−^] following an additional 1 h of moderate-intensity exercise with a 60 s end-sprint compared to pre-exercise values. Since the increase in muscle [NO_3_^−^] after NO_3_^−^ supplementation declines during exercise [9] and increases rapidly (within 30 min) following acute NO_3_^−^ ingestion [17], an in-exercise NO_3_^−^ top-up bolus after short-term NO_3_^−^ supplementation may maintain local increases in muscle [NO_3_^−^] and [NO_2_^−^]. These changes may increase muscle NO synthesis during skeletal muscle contraction [2,8] and elicit important physiological responses, such has enhanced skeletal muscle calcium handling [52,53], culminating in improved exercise performance. Further research is therefore required to assess whether the NO_3_^−^ supplementation strategy administered in the current study can impact skeletal muscle [NO_3_^−^] and [NO_2_^−^] dynamics during exercise, and the intramuscular mechanisms that underpin its ergogenic effect on longer duration endurance exercise.

## 5. Conclusions

The current study investigated the effects of short-term NO_3_^−^ supplementation, with NO_3_^−^ ingested 2 h prior to and 1 h into 2 h moderate-intensity cycling exercise terminating with a 60 s maximal-intensity end-sprint on day 7 of supplementation, on plasma [NO_3_^−^] and [NO_2_^−^], muscle [HHb] kinetics and end-sprint performance. Compared to pre-exercise values after NO_3_^−^ supplementation, consumption of an in-exercise NO_3_^−^ top-up elicited a further increase in plasma [NO_3_^−^] and attenuated the decline in plasma [NO_2_^−^] typically reported when NO_3_^−^ is only consumed pre-exercise. Short-term NO_3_^−^ supplementation combined with ingestion of a NO_3_^−^ top-up during exercise speeded muscle [HHb] kinetics during the initial stages, and improved mean power output over the final 30 s, of the end-sprint. The findings of the current study reveal a NO_3_^−^ supplementation strategy with the potential to enhance aspects of performance during prolonged endurance exercise in recreationally active individuals.

## Figures and Tables

**Table 1:** Plasma nitrate and nitrite concentrations at rest and following 2 h moderate-intensity exercise and a 60 s maximal-intensity end-sprint after ingestion of nitrate-rich and nitrate-depleted beetroot powder.

	Placebo	Beetroot
Plasma [NO_3_^−^]		
Resting baseline (μM)	59 ± 10	300 ± 73 *
Post exercise (μM)	73 ± 15	562 ± 89 *#
Plasma [NO_2_^−^]		
Resting baseline (μM)	144 ± 44	280 ± 58 *
Post exercise (μM)	135 ± 70	228 ± 63 *

Data are presented as group mean ± SD. * = higher than placebo (*p* < 0.001). # = higher than resting baseline (*p* < 0.05). Placebo, nitrate-depleted beetroot powder; beetroot, nitrate-rich beetroot powder.

**Table 2:** Time to peak power output, peak power output and mean power output variables during a maximal-intensity 60 s end-sprint following 2 h moderate-intensity exercise after ingestion of nitrate-rich and nitrate-depleted beetroot powder.

	Placebo	Beetroot
Time to peak power output (s)	5.8 ± 1.5	6.2 ± 0.9
Mean power output 0–60 s (W)	472 ± 73	495 ± 67
Mean power output 0–30 s (W)	578 ± 132	600 ± 116
Mean power output 30–60 s (W)	365 ± 41	390 ± 38 *

Data are presented as group mean ± SD. * = higher than placebo (*p* < 0.01). Placebo, nitrate-depleted beetroot powder; beetroot, nitrate-rich beetroot powder.

**Table 3:** Tissue saturation index during 2 h moderate-intensity exercise terminating in a maximal-intensity 60 s end-sprint after ingestion of nitrate-rich and nitrate-depleted beetroot powder.

	Placebo	Beetroot
	Moderate-intensity exercise	
Baseline (%)	66 ± 3	69 ± 5
20 min (%)	64 ± 4	66 ± 7
40 min (%)	65 ± 5	66 ± 7
60 min (%)	65 ± 4	65 ± 7
80 min (%)	65 ± 4	64 ± 7
100 min (%)	65 ± 5	65 ± 7
	End-sprint	
Baseline (%)	65 ± 5	67 ± 7
30–60 s (%)	59 ± 3	61 ± 5

Data are presented as group mean ± SD. Placebo, nitrate-depleted beetroot powder; beetroot, nitrate-rich beetroot powder.

## Data Availability

Data are available from the corresponding author upon reasonable request.
